# Investigating the Relationship Between Hopelessness, Alexithymia, Mind Wandering, Rumination, and Clinical Features in Patients with Bipolar Disorder

**DOI:** 10.3390/brainsci15060596

**Published:** 2025-06-02

**Authors:** Andrea Aguglia, Tommaso Cerisola, Martina Rimondotto, Simona Iannini, Francesco Bruni, Francesca Bigiotti, Alessandra Costanza, Mario Amore, Andrea Amerio, Gianluca Serafini

**Affiliations:** 1 Department of Neuroscience, Rehabilitation, Ophthalmology, Genetics, Maternal and Child Health, Section of Psychiatry, University of Genoa, Largo Paolo Daneo 3, 16132 Genoa, Italy; tomceri8@gmail.com (T.C.); m.rimondotto@gmail.com (M.R.); simona.iannini94@gmail.com (S.I.); dott.brunifrancesco@yahoo.com (F.B.); francescabigiotti@gmail.com (F.B.); mario.amore@unige.it (M.A.); andrea.amerio@unige.it (A.A.); gianluca.serafini@unige.it (G.S.); 2 IRCCS Ospedale Policlinico San Martino, Largo Rosanna Benzi 10, 16132 Genoa, Italy; 3 Department of Psychiatry, Faculty of Medicine, Geneva University (UNIGE), 24 Rue du General-Dufour, 1211 Geneva, Switzerland; alessandra.costanza@unige.ch; 4 Department of Psychiatry, Faculty of Biomedical Sciences, University of Italian Switzerland (USI), via Buffi 13, 6900 Lugano, Switzerland

**Keywords:** bipolar disorder, hopelessness, comorbidity, alexithymia, rumination, mind wandering

## Abstract

Background/Objectives: The understanding of the mechanisms involved in the etiopathogenesis and maintenance of Bipolar Disorder (BD) should be a priority to identify potential early clinical markers that could help in improving treatment strategies and prevention. The aim of this study was to investigate the potential correlation between hopelessness, alexithymia, mind wandering, and rumination in patients with a primary diagnosis of BD, evaluating whether these psychopathological aspects could negatively affect bipolar illness. Methods: A semi-structured interview was used to collect sociodemographic and clinical characteristics. Several psychometric tools were administered: the Beck Hopelessness Scale; Toronto Alexithymia Scale; Rumination Response Scale; Mind Wandering Questionnaire; Mind Wandering: Deliberate; Mind Wandering: Spontaneous; and the Daydreaming Frequency Scale. Results: Patients with high levels of hopelessness have a greater number of psychiatric and medical comorbidities and are more frequently on polypharmacotherapy. Additionally, patients with high levels of hopelessness show a greater likelihood of having attempted suicide during their lifetime. The presence of alexithymia is associated with longer hospitalization and psychiatric comorbidities. Higher levels of rumination correlate with a greater number of psychiatric and medical comorbidities, and with the presence of residual symptoms. Mind wandering is associated with the presence of medical comorbidities and residual symptoms. Conclusions: Hopelessness, alexithymia, mind wandering, and rumination should be identified as important proxies of impaired subjective well-being that should be carefully monitored because they could further worsen the clinical course of BD and suicidal risk in this vulnerable population.

## 1. Introduction

Bipolar Disorder (BD) is a severe, chronic, and cyclic psychiatric disorder characterized by recurrent affective episodes [1], affecting approximately 2–3% of the general population and representing a leading cause of disability worldwide [2]. 

Patients with BD have a higher prevalence of both psychiatric and medical comorbidities compared to the general population [3], and this could represent a potential risk factor contributing to the frequent use of long-life complex pharmacological treatment [4,5,6]. A primary diagnosis of BD is also associated with premature mortality, due to higher rates of suicidal behaviors and cardio-vascular–metabolic comorbidities [7,8,9]. BD can be differentiated into type I and II, according to the presence of manic and hypomanic episodes, respectively. Based on the Diagnostic and Statistical Manual of Mental Disorders, Fifth Edition, Text Revision (DSM 5-TR), the distinction between manic and hypomanic episodes considers different clinical aspects, such as a duration of a minimum of seven and four days for manic and hypomanic episodes, respectively; a negative impact on functioning; and the presence of psychotic, confusional, or pantoclastic symptoms in a manic episode. It should be noted that hypomanic and major depressive episodes can be present in both disorders. Both BD type I and II have significant negative impacts on quality of life and require ongoing personalized clinical management.

Understanding the mechanisms involved in the etiopathogenesis and maintenance of BD should be a priority in order to identify potential early clinical markers that could help in improving treatment and prevention strategies. Therefore, a deeper understanding and analysis of particular clinical features, such as alexithymia, hopelessness, mind wandering, and rumination could be helpful for clinicians, especially when assessing suicidal risk and comorbidities. Hopelessness, defined as a subjective emotion characterized by feelings of powerlessness and pessimism about the future, is a transdiagnostic clinical dimension accepted as an indicator of high suicide risk not only in neurological and psychiatric patients but also in the general population [10,11,12]. A study on outpatients with a primary diagnosis of BD suggested that hopelessness may be considered a trait factor conferring greater vulnerability for suicidal behaviors [13] and, recently, insomnia symptomatology has been considered a potential mediator of this correlation [14]. On the other hand, alexithymia is a psychological condition characterized by difficulties in understanding and describing one’s emotions and distinguishing feelings from bodily arousal signals. High levels of alexithymia lead to a limited capacity for imagination and symbolic thinking, with negative consequences on general and social functioning in BD, due to objective difficulties in the expression of emotions through symbols or metaphors, contributing to worsening outcomes and a greater risk of suicidality [15,16,17]. Recently, an association between childhood emotional abuse and alexithymia with bipolar symptoms and suicidal ideation, mediated by emotional dysregulation, has been demonstrated [18,19,20]. In addition, rumination is defined as a response to a depressed mood that involves focusing on the possible meanings, causes, and consequences of depressive symptoms passively and repeatedly, tending to keep people fixed on their problems and feelings without acting [21,22,23]. Rumination worsens emotional dysregulation and affective lability in all mood bipolar recurrences, aggravating both depressive and (hypo)manic phases and also contributing to suicidal behaviors and potentially dangerous behaviors, respectively [22,24]. Finally, mind wandering is defined as a moment when attention shifts from the perceptual world (i.e., the ongoing task) to focus on the internal world (one’s thoughts and feelings) [25]. Both rumination and mind wandering are associated with negative emotions, compromising patients’ mental well-being and leading to maladaptive mental states as low self-esteem and negative cognitive loops which could further elicit or worsen suicidal ideation [26,27,28,29].

Therefore, the aim of this study was to investigate the potential correlation between hopelessness, alexithymia, mind wandering, and rumination in patients with a primary diagnosis of BD, evaluating whether these psychopathological aspects could negatively affect bipolar illness. Our main hypothesis is that feelings of hopelessness, alexithymia, mind wandering, and rumination may, indeed, predict a worsening of clinical and psychopathological bipolar course, with a higher prevalence of medical or psychiatric comorbidities and suicidal behaviors. 

## 2. Materials and Methods

### 2.1. Study Design and Participants

A cross-sectional study was conducted on one hundred and seventy-one patients with a primary diagnosis of BD, according to the criteria of the DSM 5-TR [1]. All the participants were consecutively admitted to the Section of Psychiatry, Department of Neuroscience, Rehabilitation, Ophthalmology, Genetics, Maternal and Child Health (DiNOGMI), IRCCS Ospedale Policlinico San Martino, University of Genoa (Italy), from September 2022 to April 2024.

An in-depth description of the study was given to all potential participants, explaining the study objectives and procedures in detail with the opportunity to ask questions about the goals of the study. 

The study was designed in agreement with the guidelines from the Declaration of Helsinki, as revised in 2013 [30], and was approved by the Local Research Ethical Committee. 

### 2.2. Assessment

A semi-structured interview, used in previously published research articles [6,31,32], was administered to collect the following sociodemographic and clinical characteristics: age, gender, duration of illness and hospitalization, age of onset, presence of psychotic and residual symptoms, suicidal ideation, current or lifetime suicidal attempts, psychiatric and medical comorbidities, use of complex pharmacological treatment, and ongoing psychotherapy treatment.

Furthermore, each participant completed a battery of psychometric tools, including the Beck Hopelessness Scale (BHS), Toronto Alexithymia Scale (TAS-20), Ruminative Response Scale (RRS-22), and Mind Wandering Questionnaire (MWQ). The validated Italian versions of each scale were used for this study, and each psychometric tool was presented and explained to the patients. They were informed that the data gathered with these tools could be useful in recognizing a subgroup of patients who could potentially benefit from a specific non-pharmacological personalized treatment approach.

- The BHS is a 20-item self-administered questionnaire designed to measure hopelessness, its different dimensions, and more specifically, feelings about the future, loss of motivation, and expectations [33].
- The TAS-20 is a self-report scale consisting of 20 items measuring difficulty in identifying feelings, including the differentiation between emotional states and bodily sensations; difficulty in describing feelings to others; and a constricted imaginative process with an externally oriented cognitive style. Scores equal to or greater than 61 indicate the presence of alexithymia [34].
- The RRS is a 22-item self-administered questionnaire commonly used to evaluate ruminative tendencies, consisting of three factors: brooding (e.g., ‘[how often do you] think “why do I have problems other people don’t have?”’), depression (e.g., ‘[how often do you] think about how sad you feel’), and self-reflection (e.g., ‘[how often do you] go someplace alone to think about your feelings’) [35,36]. Items are rated on a scale ranging from 1 to 4 (almost never, sometimes, often, and almost always), with higher scores indicating worse ruminative symptoms.
- The MWQ is a 5-item scale designed to assess the frequency and propensity of mind wandering, both in its deliberate or spontaneous form. Each item is rated on a Likert scale from 1 (almost never) to 6 (almost always) [37]. The items on this scale are written in simple language to allow for a good applicability adolescents as well as adults [38].

### 2.3. Statistical Analysis

Statistical analyses were performed using R software version 4.3.1 (V.4.3.1; R package meta, R Foundation for Statistical Computing, Vienna, Austria). The statistical significance was set at *p* < 0.05. Continuous and categorical variables were presented as means and standard deviations (SDs) or frequency and percentage, respectively.

Linear correlations between sociodemographic data and the results of the clinical assessments were evaluated using Spearman’s Rank Correlation. The patients were also divided into two subgroups depending on their specific bipolar diagnosis (BD type I and II). To further investigate a potential correlation between the variables of interest and patients’ diagnoses, two-sample *t*-test analysis was performed for continuous variables. The Smirnov–Grubbs test was performed to confirm the absence of outliers. As this was an exploratory study aimed at identifying potential associations, no correction for multiple comparisons was applied. The findings should be considered hypothesis-generating and warrant further confirmation in future longitudinal studies using larger samples and correction procedures.

## 3. Results

### 3.1. Sociodemographic and Clinical Characteristics

The total sample included 171 patients with a primary diagnosis of BD, of which 86 (50.3%) were females. The mean age was 48 years (SD ± 14). A total of 70 (40.9%) patients had a diagnosis of BD type I, whereas 101 (59.1%) had a diagnosis of BD type II. For all patients, the mean age of onset of BD was 27 years (SD ± 11), while the mean duration of illness was 21 years (SD ± 13). The mean duration of hospitalization was 15 days (SD ± 4.4). Twenty-seven patients (15.8%) showed lifetime psychotic symptoms, while forty-one patients (24.0%) had lifetime residual symptoms. 

Most patients had psychiatric or medical comorbidities. A total of 90 patients (52.6%) had psychiatric comorbidities and 100 patients (58.5%) had medical comorbidities. About half of the patients were taking at least four medications (*n* = 69, 40.4%), whereas only 51 of them (29.8%) were undergoing non-pharmacological treatment. Finally, 21 patients (12.3%) had been admitted to the psychiatric ward for a suicide attempt and 70 patients (41.0%) reported a lifetime suicide attempt. All the sociodemographic and clinical characteristics are summarized in Table 1. 

### 3.2. Correlation Analysis Between Hopelessnesss, Alexithymia, Rumination, Mind Wandering, and Sociodemographic and Clinical Characteristics

In our study, no significant correlation was found between the psychopathological and sociodemographic characteristics of our patients, whereas several positive associations with their novel clinical dimensions were observed. Patients with higher levels of hopelessness had more psychiatric (*p* = 0.04) and medical comorbidities (*p* = 0.03). Furthermore, these patients were more likely to have complex polypharmacotherapy (*p* = 0.02). On the other hand, the presence of alexithymia was positively associated with longer durations of hospitalization, particularly in patients with higher scores on the difficulties in externally oriented thinking subscale (*p* = 0.03), and psychiatric comorbidities, particularly in patients with higher scores for difficulties in identifying emotions (*p* = 0.01).

Specifically regarding rumination, this clinical dimension was significantly associated with the presence of residual symptoms (*p* = 0.04) and more psychiatric (*p* = 0.01) and medical comorbidities (*p* = 0.04), especially in those with higher scores in the depression subscale (*p* = 0.04). Furthermore, patients who scored higher in the RRS depression subscale were more likely to take at least four medications (*p* = 0.03). Moreover, a significant association between mind wandering and the presence of medical comorbidities (*p* = 0.02) was observed, particularly when it was more frequent (*p* = 0.04). Patients who scored higher in the subscale measuring deliberate mind wandering showed more residual symptoms (*p* = 0.03), especially when mind wandering was more frequent as measured by the DDFS (*p* = 0.02). Finally, no significant correlation was observed between suicidal behaviors (suicidal ideation and current/lifetime suicide attempts) and rumination or mind wandering. The only significant correlation was found between lifetime suicide attempts and higher levels of hopelessness (*p* = 0.01) (see Table 2). 

### 3.3. Difference in Clinical Dimensions Investigated Between BD Type I and II

In our study, considering the difference between BD type I and II, no statistically relevant differences in rumination, mind wandering, or alexithymia were found. However, a feeling of hopelessness was more prevalent in patients with BD type II (Table 3).

## 4. Discussion

The findings of this research provide insights critical to better understanding the course of BD, its psychopathological manifestations, and the impact of several clinical dimensions not considered in the diagnostic criteria of the DSM 5-TR. Hopelessness, alexithymia, rumination, and mind wandering were investigated and correlated with the sociodemographic and clinical characteristics of patients with a primary diagnosis of BD. As expected, an equal gender distribution, an average age of 48 years, and a significant prevalence of psychiatric and medical comorbidities were found. Most patients were on complex polypharmacotherapy, with a notable portion also engaged in non-pharmacological treatment. Finally, a considerable number of patients had been previously hospitalized following a suicide attempt.

In the literature, it is well known that feelings of hopelessness are accepted as a transnosographic and transdiagnostic measure of increased risk for suicidal behaviors in psychiatric patients and the general population [39]. In our sample, a strong association was observed between high levels of hopelessness and the presence of psychiatric and medical comorbidities. The International Society for Bipolar Disorders (ISBD) task force on suicide summarized all the sociodemographic and clinical factors potentially associated with increased suicidal behaviors [40]. In particular, the presence of comorbid anxiety disorders, substance use disorders, or Cluster B personality disorders, especially borderline personality disorder, are considered risk factors for suicidal behaviors. Furthermore, these psychiatric comorbid conditions are known to exacerbate the severity of BD, potentially reflecting a more challenging and destabilizing clinical course [32,41,42]. From this point of view, it is not surprising that the feelings of hopelessness were notably higher in bipolar patients taking at least four medications, potentially indicative of a more difficult therapeutic challenge. Though direct evidence from the literature is limited, a greater pharmacological burden may contribute to patients’ feelings of despair regarding their psychopathological condition of mood instability. The exact causality remains unclear, but the literature suggests that comorbid conditions may either signal a more severe bipolar picture or contribute to increased ruminative thinking, which is closely linked to suicidal ideation [43,44]. For example, patients with comorbid anxiety disorders may experience heightened ruminative thoughts centered on suicidal behavior, while substance use disorders may amplify impulsivity, further increasing risk [45,46]. Additionally, prolonged psychiatric hospitalization was associated with higher levels of alexithymia, particularly in the externally oriented thinking (EOT) domain. This may reflect the impact of protracted inpatient care on patients’ socioemotional functioning [47].

Rumination and mind wandering were positively correlated with psychiatric and medical comorbidities, as well as residual symptoms. These transdiagnostic phenomena are increasingly recognized as key contributors to the co-occurrence of symptoms across disorders, particularly in mood disorders [48,49]. Excessive rumination, especially, has been identified as a common feature in both depressive and manic episodes [22,50], suggesting its pivotal role in BD. While studied less extensively in BD compared to unipolar depression, the interplay between rumination and hopelessness warrants further attention. Both constructs appear to perpetuate a vicious cycle, amplifying negative effects and hindering emotional regulation. Therefore, enhancing patients’ capacity to regulate intrusive and ruminative thoughts may hold promise for preventing both depressive and manic episodes [51,52]. Recently, the number of previous suicide attempts was significantly correlated with scores for reflective ponderings, brooding, and global rumination in patients with BD. Furthermore, hopelessness and rumination were correlated [53].

Although no direct correlations between rumination or mind wandering and suicidal ideation or suicide attempts were observed in this study, patients with higher levels of hopelessness were significantly more likely to have attempted suicide. This aligns with the existing literature that identifies hopelessness as a transdiagnostic measure associated with increased suicide risk [12,53,54]. Hopelessness exacerbates feelings of pessimism about the future, serving as an independent factor for increased suicide risk, and patients with a history of suicide attempts exhibit higher hopelessness scores; furthermore, they often present comorbid anxiety disorders [55]. Interestingly, patients with BD type II demonstrated higher levels of hopelessness compared to those with BD type I. This is quite expected, because patients with a primary diagnosis of BD type II usually reported more severe and longer major depressive episodes, presenting unique challenges in the evaluation and management of depression within this subtype, higher antidepressant usage, and an unstable illness course [56,57]. Furthermore, major depressive episodes are usually associated with comorbidities (especially in BD type II), with greater disability and a worse quality of life [58] often accompanied by elevated hopelessness and strongly linked to increased suicidal behavior [59]. 

The relationship between insight, hopelessness, and suicidal risk has also been explored in the literature, particularly within the proposed “insight-demotivation-depression-suicidality” syndrome [60]. In BD, the interplay between hopelessness, psychiatric comorbidities, and emotional dysregulation may reflect a similar phenomenon, further underscoring the importance of targeted interventions. Chronicity, global impairment, and the persistence of residual symptoms despite comprehensive pharmacological treatment highlight the need for multifaceted therapeutic approaches [61,62]. It is well known that suicide risk is notably higher in depressive mixed states of BD compared to manic, depressive, or euthymic phases [63,64,65]. From a clinical perspective, our findings underline the need for greater attention to residual symptoms, suicidal behaviors, and psychiatric or medical comorbidities in patients with BD. As a matter of fact, these clinical factors lead not only to a worse outcome in terms of quality of life and functioning, but also to higher reported levels of hopelessness, alexithymia, mind wandering, and rumination. These patients often represent more complex clinical cases, characterized by frequent hospitalizations, a more severe illness trajectory, and an increased risk of suicidal behaviors [12,22,32,40,53]. Despite the clinical relevance of these psychopathological dimensions, current guidelines do not provide specific recommendations for managing these transdiagnostic features in patients with BD. Our findings highlight the importance of structured, personalized interventions to treat these psychological vulnerabilities. For example, in cases of rumination and hopelessness, the use of antidepressants could be avoided in favor of specific non-pharmacological interventions, such as structured psychoeducation and enhanced family support, to improve patient outcomes and functional recovery. These insights support a shift toward individualized care in BD, moving beyond syndromic classifications and focusing on the broader psychopathological profile of each patient.

## 5. Limitations

The present study was preliminary and exploratory due to the limited literature on this topic. Despite the clinical relevance of our findings, this study has certain limitations that should be discussed. First, the study had a cross-sectional design, limiting statements regarding causality or the directionality of the associations observed due to the lack of follow-up data. Further longitudinal studies are warranted to clarify the temporal relationships between hopelessness, alexithymia, rumination, mind wandering, and clinical outcomes in individuals with BD. Secondly, the novel clinical dimensions, such as hopelessness, alexithymia, rumination, and mind wandering, were evaluated by self-rated scales, with potential response biases like social desirability and recall bias due to the intrinsic nature of self-reporting. Future research should consider incorporating multi-method assessment strategies, such as clinician-administered psychometric tools, observer-rated scales, and biological or behavioral measures, to improve the reliability and validity of the findings. Third, our sample was relatively small, including only inpatients from a single psychiatric unit and considering only severe cases, which may limit the generalizability of the results to the broader bipolar population, particularly outpatients or individuals with less severe presentations. Future studies should aim to include larger and more heterogeneous samples to enhance external validity and provide more representative data. Additionally, the study involved multiple statistical comparisons without applying correction methods such as Bonferroni adjustment or False Discovery Rate control. While this approach aligns with the exploratory intent of the analysis, it increases the risk of type I errors. These results should be interpreted with caution and validated in future longitudinal research using appropriate designs and appropriate statistical correction strategies.

## 6. Conclusions

The significant correlation between novel clinical dimensions, comorbidities, and suicidal behaviors highlights the critical need for clinicians to assess and address these factors in their daily clinical practice. Importantly, hopelessness, alexithymia, mind wandering, and rumination should be identified as important proxies of impaired subjective well-being that should be carefully monitored because they could further worsen the clinical course of BD and suicidal risk in this vulnerable population. 

Further, longitudinal studies are needed to replicate our preliminary and exploratory results and may further help to ameliorate the destabilizing course of BD, implement appropriate and personalized non-pharmacological strategies, and discover more solid links, affirming the importance of these factors in order to increase the quality of life, extend the psychophysical well-being, and promote the functional recovery of patients with BD.

## Figures and Tables

**Table 1:** Sociodemographic and clinical characteristics of total sample.

N (%) or Mean ± SD	Total Sample (*n* = 171)
Sociodemographic characteristics	
Gender, female, N (%)	86 (50.3)
Current age in years	48 ± 14
Marital status Single Married Divorced/separated Widowed	116 (68.3) 29 (17.0) 24 (14.3) 1 (0.4)
Educational level in years	11.6 ± 3.3
Italian nationality	151 (88.4)
Occupational status, employed	69 (40.2)
Living with Alone Family Residence	62 (36.6) 105 (61.3) 4 (2.1)
Clinical characteristics	
Length of hospitalization in days	15 ± 4.4
Age onset in years	27 ± 11
Illness duration in years	21 ± 13
Suicidal ideation	76 (44.7)
Suicide attempts Current Lifetime	21 (12.3) 70 (41.0)
At least one psychiatric comorbidity	90 (52.6)
At least one medical comorbidity	100 (58.5)
Lifetime psychotic symptoms	27 (15.8)
Lifetime residual symptoms	41 (24.0)
Complex polypharmacotherapy	69 (40.4)
Non-pharmacological treatment	51 (29.8)

**Table 2:** Linear correlation between clinical characteristics and psychopathological dimensions investigated.

*Rho*		Length of Hospitalization	Psychiatric Comorbidity	Medical Comorbidity	Complex Pharmacotherapy	Residual Symptoms	Lifetime Suicide Attempts
	*p*						
BHS tot		−0.0908 0.85	0.151 0.0419	0.167 0.025	0.172 0.022	0.094 0.14	0.245 <0.01
RRS tot		0.0074 0.46	0.213 <0.01	0.149 0.0405	0.066 0.221	0.151 0.0404	0.02 0.41
RRS depression		−0.016 0.57	0.0784 0.18	0.148 0.0421	0.156 0.034	0.113 0.096	0.022 0.40
RRS reflection		−0.0207 0.59	0.0507 0.27	0.134 0.0588	0.0194 0.41	0.11 0.10	0.09 0.15
TAS dif		−0.133 0.93	0.196 <0.01	−0.0682 0.78	0.0959 0.13	−0.115 0.91	0.127 0.07
TAS eot		0.162 0.0316	−0.109 0.55	−0.129 0.93	−0.003 0.502	−0.073 0.79	0.025 0.38
MWQ tot		−0.00009 0.54	0.0766 0.18	0.182 <0.01	0.071 0.203	0.097 0.13	0.056 0.26
MWD tot		−0.0785 0.81	−0.0135 0.56	0.105 0.11	0.084 0.16	0.165 0.0279	0.104 0.12
DDFS tot		−0.0192 0.58	0.0584 0.24	0.148 0.0418	0.061 0.24	0.183 <0.01	0.057 0.26

BHS: Beck Hopelessness Scale; RRS: Ruminative Response Scale; TAS: Toronto Alexithymia Scale; MWQ: Mind Wandering Questionnaire; MW.D: Mind Wandering Deliberate; MW.S: Mind Wandering Spontaneous; DDFS: Daydreaming Frequency Scale.

**Table 3:** Difference in clinical dimensions investigated between BD type I and II.

Mean ± SD	BD Type I	BD Type II	*p*
BHS tot	5.18 ± 4.12	7.41 ± 5.55	0.02
RRS tot	33.91 ± 22.47	38.98 ± 27.31	0.29
RRS depression	9.67 ± 5.19	8.86 ± 5.04	0.14
RRS reflection	7.96 ± 4.35	8.86 ± 5.04	0.84
RRS brooding	16.61 ± 10.07	19 ± 12.49	0.27
TAS tot	41.31 ± 20.21	43.39 ± 22.53	0.61
TAS level	4.26 ± 6.05	4.41 ± 5.78	0.89
TAS ddf	13.07 ± 3.96	14.52 ± 5.28	0.11
TAS dif	16.61 ± 7.69	16.98 ± 7.55	0.79
TAS eot	26.33 ± 13	26.07 ± 12.79	0.91
MWQ tot	11.91 ± 8.37	12.36 ± 8.98	0.78
MWD tot	10.37 ± 8.31	9.14 ± 6.86	0.39
MWS tot	11.35 ± 7.98	10.61 ± 8.67	0.64
DDFS tot	21.72 ± 16.19	21.36 ± 16.06	0.91

BD type I: Bipolar Disorder type I; BD type II: Bipolar Disorder type 2; BHS: Beck Hopelessness Scale; RRS: Ruminative Response Scale; TAS: Toronto Alexithymia Scale; MWQ: Mind Wandering Questionnaire; MW.D: Mind Wandering Deliberate; MW.S: Mind Wandering Spontaneous; DDFS: Daydreaming Frequency Scale.

## Data Availability

The data presented in this study are available on request from the corresponding author. The data are not publicly available due to privacy/ethical restrictions.

## Abbreviations

The following abbreviations are used in this manuscript:

BD type I	Bipolar Disorder type I
BD type II	Bipolar Disorder type II
BHS	Beck Hopelessness Scale
RRS	Ruminative Response Scale
TAS	Toronto Alexithymia Scale
MWQ	Mind Wandering Questionnaire
MWD	Mind Wandering Deliberate
MWS	Mind Wandering Spontaneous
DDFS	Daydreaming Frequency Scale
SD	Standard deviation
